# A Single Center Study on the Risks of Peri-Intervention Stroke in Thoracic Endovascular Aortic Repair (TEVAR) and Endovascular Abdominal Aortic Repair (EVAR)

**DOI:** 10.3390/jcdd9010010

**Published:** 2022-01-03

**Authors:** Jirayoot Chusooth, Chanon Kongkamol, Ruedeekorn Suwannanon, Dhanakom Premprabha, Voravit Chittithavorn, Pannawit Benjhawaleemas, Hutcha Sriplung, Pornchai Sathirapanya

**Affiliations:** 1 Division of Neurology, Department of Internal Medicine, Faculty of Medicine, Prince of Songkla University, Hat Yai 90110, Thailand; jirayoot018@gmail.com; 2 Department of Family and Preventive Medicine, Faculty of Medicine, Prince of Songkla University, Hat Yai 90110, Thailand; chanon.kon@psu.ac.th; 3 Department of Radiology, Faculty of Medicine, Prince of Songkla University, Hat Yai 90110, Thailand; sruedeekorn@gmail.com; 4 Department of Surgery, Faculty of Medicine, Prince of Songkla University, Hat Yai 90110, Thailand; dhanakom@msn.com (D.P.); cvoravit@medicine.psu.ac.th (V.C.); 5 Department of Anesthesiology, Faculty of Medicine, Prince of Songkla University, Hat Yai 90110, Thailand; ccprecha@gmail.com; 6 Epidemiology Unit, Faculty of Medicine, Prince of Songkla University, Hat Yai 90110, Thailand; hutcha.s@medicine.psu.ac.th

**Keywords:** aorta, intervention, stroke, complication

## Abstract

(1) Background: The risk factors of peri-intervention stroke (PIS) in thoracic endovascular aortic repair (TEVAR) and endovascular abdominal aortic repair (EVAR) are different. This study aimed to compare the risks of PIS in both interventions. (2) Methods: Patients who had suffered a PIS related to TEVAR or EVAR from January 2008 to June 2015 in Songklanagarind Hospital were selected as the cases, while patients who had not suffered PIS were randomly selected to create a 1:4 case: control ratio for analysis. The associations between the factors from pre- to post-intervention and PISs in TEVAR or EVAR cases were analyzed by univariable analysis (*p* < 0.1). The independent risks of PIS were identified by multivariable analysis and presented in odds ratios (*p* < 0.05). (3) Results: A total of 17 (2.2%) out of 777 patients who had undergone TEVAR or EVAR experienced PIS, of which 9/518 (1.7%) and 8/259 (3.1%) cases were in TEVAR and EVAR groups, respectively. PIS developed within the first 24 h in nine (52.9%) cases. Large vessel ischemic stroke or watershed infarctions were the most common etiologies of PIS. The independent risks of PIS were the volume of intra-intervention blood loss (1.99 (1.88–21.12), *p* < 0.001) in the TEVAR-related PIS, and intervention time (2.16 (1.95–2.37), *p* = 0.010) and post-intervention hyperglycemia (18.60 (1.60–216.06), *p* = 0.001) in the EVAR-related PIS. There were no differences in the rate of PIS among the operators, intervention techniques, and status of the interventions performed. (4) Conclusion: The risks of PIS in TEVAR or EVAR in our center were different and possibly independent of the operator expertise and intervention techniques.

## 1. Introduction

There is currently a growing number of minimally invasive surgeries, for example, body tissue intervention for performing a tissue biopsy or treatment and endovascular intervention (EVI) for treating vascular pathologies. Although EVI causes less body tissue injury compared with conventional surgery, its associated vascular complications, particularly strokes, remain a critical concern pending an effective procedure for prevention. Unlike conventional open surgery, in which the variations in the levels of various tissue thrombophilic factors induced by surgical injury during or post-operation are attributed as a cause of stroke [1,2], a pre-existing atheromatic plaque plus manipulation of the device wire within the vascular lumen can lead to the increased risks of peri-intervention thromboembolism [3]. Dislodgement of an aortic atheroma caused by the intravascular manipulation of the device while performing an aortic EVI, e.g., thoracic endovascular aortic repair (TEVAR) or endovascular abdominal aortic repair (EVAR), can result in an aorto-cerebral embolism, a common etiology of peri-intervention stroke (PIS) [4,5,6]. PIS related to an aortic EVI is a serious complication [7]. As the knowledge of performing aortic EVIs has continuously progressed, further studies are needed to develop effective strategies for PIS prevention [8]. While most studies to date have focused on the intervention techniques, devices, supplementary procedures for PIS prevention, and rate of PIS in TEVAR [8,9,10,11,12,13,14], only a few have investigated the risks of PIS in EVAR. Hence, the current study aimed to study the rates and the significant risks of PIS in TEVAR and EVAR during the pre-, intra- and post-intervention periods. Despite the limited number of study cases and a single-center study, in which sharing of operators’ expertise and techniques is common, we believe that our study is useful in demonstrating the true risks of PIS in each intervention.

## 2. Materials and Methods

### 2.1. Study Population and Setting

All patients aged ≥18 years who experienced PIS related to either TEVAR or EVAR from January 2008 to June 2015 in Songklanagarind Hospital, a tertiary care and medical teaching hospital, were enrolled. Patients who did not acquire PIS as a complication of either intervention (non-PIS) were randomly selected from the hospital medical records using the website https://www.random.org (accessed on 5 November 2021) to create a 1:4 ratio of PIS to non-PIS cases. We excluded all patients with a history of aortic trauma, severe head injury, serious multiple injuries, recent stroke (≤1 month prior to either intervention), cardio-pulmonary arrest before the intervention began, bleeding diathesis, or intra-intervention death before a stroke could be diagnosed. Emergency computerized tomography (CT) scans of the brain (CT brain) combined with CT angiography (CTA) were performed for diagnosis of a PIS when the hospital stroke team was notified. Reports of cerebral infarction including the cerebrovascular territories involved on the CT brains were verified by a clinically blinded neuroradiologist.

### 2.2. Definitions of Terms

Peri-intervention stroke (PIS) was defined as a stroke that developed at any time point from the start of either intervention to 30 days post-intervention. Since there is no specific time window after an intervention defined for PIS, the 30-day time span was applied following the accepted definition of peri-operative stroke (POS) in a conventional surgery [15,16,17].

Pre-intervention cardiovascular risks such as hypertension (HT), diabetes mellitus (DM), dyslipidemia (DLP), or atrial fibrillation (AF), were the past illnesses identified from the hospital computerized medical records or diagnosed at the time of patient presentation.

Hypotension was defined as a systolic blood pressure (SBP) < 90 mmHg or the requirement of one or more inotropic drugs to maintain an acceptable blood pressure [1].

Hyperglycemia was defined as a random blood sugar level of >250 mg/dL [18].

Hypoglycemia was diagnosed as a blood sugar level < 70 mg/dL, based on the criteria defined by the American Diabetes Association (ADA) [19].

Respiratory failure was defined as a condition for which prolonged assisted respiration (>14 days) was required after completion of either intervention, or where endotracheal re-intubation and mechanical respiration were indicated after an initially successful extubation post-intervention [20].

The grading of aortic atheroma thickness was as follows: grade I: smooth and continuous aortic intimal surface; grade II: intimal thickness of 3 to 5 mm; grade III: atheroma protruding <5 mm into the aortic lumen; grade IV: ulcerative or pedunculated atheroma protruding >5 mm into the aortic lumen [21]. The presence of an aortic arch atheroma and grading, as well as the location and grade of the maximal aortic atheroma thickness, were verified by a clinically blinded cardiovascular radiologist.

### 2.3. Data Collection

The study data were collected from the PIS and non-PIS cases who underwent TEVAR or EVAR and were analyzed separately. The collected data included patient demographics, underlying cardiovascular diseases, current antithrombotic drugs used (antiplatelet(s) and/or anticoagulant(s)) before the interventions, AF, the presence of aortic arch atheroma and its thickness grade, location and grade of the maximal aortic atheroma thickness, the diagnosis of aortic diseases and complications which were confirmed by the cardiovascular radiologist, mode of intervention performed (i.e., emergency vs. elective), method of anesthesia provided, etiology of PIS (i.e., ischemic or hemorrhagic PIS, and large or small cerebral vessel occlusion if ischemic PIS was diagnosed), estimated volume of intra-intervention blood loss, intervention time, intervention technique (e.g., coverage of left subclavian artery (LSCA), supra-aortic vascular revascularization, and chimney shunt used), intra- and post-intervention AF, hypotension, hyperglycemia, hypoglycemia, and post-intervention respiratory failure. A comparison of the total intensive care unit (ICU) admission days and total hospitalization days between the PIS and non-PIS patients was carried out. The stroke outcomes at discharge were assessed by the modified Rankin score (mRs), in which a score of 0–2 was considered favorable and 3–6 was considered unfavorable.

### 2.4. Statistical Analysis

This study analyzed the significant risks of PIS in TEVAR or EVAR separately. Descriptive statistics including the number and percentage, mean ± standard deviation (SD), and median with interquartile range (IQR) were used. An unpaired t-test or Wilcoxon rank sum test were used for continuous variables and Fisher’s exact test or a Chi-square test were used for categorical variables. Differences in demographic and clinical variables between the PIS and non-PIS groups were initially identified by univariable analysis (*p* < 0.1), followed by multivariable analysis of the significant independent associations, which were reported in crude odds ratios (*p* < 0.05). The R program [22] was used for statistical analysis with the “DiagnosisMed” [23], “epicalc“ [24], and “ICE“ packages [25].

### 2.5. Ethical Consideration

The Institutional Ethics Review Board of Faculty of Medicine, Prince of Songkla University approved the study protocol (EC protocol code 59-199-14-4; date of approval 29 September 2016). The study process strictly followed the guidelines of the 1964 Declaration of Helsinki and its later amendments. All personal information and identification of the study participants were completely anonymized.

## 3. Results

This study included 518 and 259 patients who underwent TEVAR and EVAR, respectively. The cases excluded were four (one TEVAR and three EVAR) intra-intervention deaths; one (EVAR) subarachnoid hemorrhage; one (TEVAR) ruptured arteriovenous malformation; and 25 (21 TEVAR and 4 EVAR) traumatic injuries of the aorta. We found 17 (2.2%) patients, 9/518 (1.7%) TEVAR-related and 8/259 (3.1%) EVAR-related PIS, who were eligible for final statistical analysis. The prevalence of PIS was highest in the first 24 h after the interventions, with nine cases diagnosed (five TEVAR- and four EVAR-related), followed by four cases (three TEVAR- and one EVAR-related) on the second day, and one case (TEVAR-related) on the third day. Another three EVAR-related PIS cases occurred on days 7 (2 cases) and 12 (1 case) post-intervention. In all PIS cases, the hospital stroke treatment team was activated and the CT brains and CTAs were performed immediately. Thirteen CT brains showed a subacute cortical cerebral infarction of a large cerebral vessel occlusion (LVO) or a watershed infarction due to globally low cerebral blood flow, while four cases showed negative results (two TEVAR- and two EVAR-related). The cerebrovascular territories involved were three cases of carotid infarction (middle cerebral artery), one case of vertebrobasilar infarction (posterior cerebral artery), and three cases of watershed infarction in TEVAR-related PIS patients, while two cases of carotid infarction (one anterior and one middle cerebral artery), three cases of vertebrobasilar infarction (two vertebral and one posterior cerebral artery), and one case of watershed infarction were found in the EVAR-related PIS patients. No intracerebral hemorrhage or spinal cord infarction cases were found in this study.

From the univariable analysis, no significant differences were found between the TEVAR-related PIS and non-PIS patients regarding the pre-intervention aortic pathology, i.e., diagnosis of aortic aneurysm or dissection, location of the pathology at the aortic arch or descending aorta, ruptured or unruptured aortic aneurysms, or site of the maximal atheroma thickness and its grade, except for the presence of aortic arch atheroma and its grade (Table 1). Similarly, no significant pre-intervention factors associated with EVAR-related PIS were found. (Table 2).

When the intra-intervention factors of TEVAR and EVAR were considered, the study results showed no significant differences between the percentage of PIS and non-PIS patients in regard to the operators who performed the interventions, modes of the intervention carried out (emergency or elective), or anesthetic method used. However, this study found that the intervention time, volume of blood loss, and hyperglycemia in the TEVAR group, and the intervention time and chimney graft used in the EVAR group, were associated with PIS in univariable analysis (*p* < 0.1) (Table 1 and Table 2).

During the post-intervention period, hyperglycemia was significantly associated with PIS in both the TEVAR and EVAR groups, while AF and respiratory failure were significant only in the EVAR group (Table 1 and Table 2). Three (two TEVAR and one EVAR) of eleven (seven TEVAR and four EVAR) post-intervention hyperglycemia cases had been diagnosed with DM, and three (two TEVAR and one EVAR) of five (one TEVAR and four EVAR) post-intervention AF cases had been diagnosed with AF before the interventions.

The independent risk factors for PIS were the volume of intra-intervention blood loss in the TEVAR-related PIS group (Table 1), and the intervention time and post-intervention hyperglycemia in the EVAR-related PIS group (Table 2). No pre-intervention patient characteristics, cardiovascular risks, aortic pathologies, operators, techniques (coverage of LSCA with revascularization or chimney graft used), or mode of intervention were independent risk factors for PIS in either intervention.

The overall in-hospital mortality rate among the PIS patients was 5/17 (29.4%) cases (three TEVAR and two EVAR cases), compared with 3/68 (4.4%) cases (two TEVAR and one EVAR cases) in the non-PIS patients. The causes of death were ventilator-associated pneumonia and severe sepsis (four cases) and massive brain edema (one case). The median (IQR) days of ICU admission was 9 days (7, 39) in the PIS patients compared with 0 days (0, 3) in the non-PIS patients (*p* < 0.001). The median (IQR) days of hospitalization in the PIS patients was 36 days (21, 56) compared with 3 days (3, 11) in the non-PIS cases (*p* < 0.001). For the stroke outcome on discharge, the median (IQR) mRS in the PIS patients was 4 (2, 6).

## 4. Discussion

The rates of PIS in TEVAR and EVAR in our study were 1.7% and 3.1%, respectively. All the PIS cases in this study were ischemic stroke. No cases of intracerebral hemorrhage or spinal cord ischemia were presented. Previous studies have reported the rates of TEVAR-related PIS ranging from 3.7 to 9.4% [4,5,26,27,28,29]. A pooled prevalence study of PIS following TEVAR of descending aortic aneurysms reported an average incidence of 4.1% (range 0–7.2%; 95% CI, 2.9–5.5%) as well [30]. Most of the PISs related to TEVAR reported in the literature were ischemic stroke, while intracerebral hemorrhage or spinal cord ischemia was less common [7,9,31,32]. However, our literature search found that studies exploring EVAR-related PIS were very limited. More than half of the PISs in our study were reported to the hospital stroke team by their attending physicians or nurses within the first day after the interventions (9/17, 52.9% (five TEVAR-related and four EVAR-related)). This is comparable to the number of PISs diagnosed within 24 h after TEVAR in a previous study [7]. Fourteen of seventeen (82.4%) PIS cases related to TEVAR or EVAR occurred within 3 days after the interventions. The high prevalence of PISs shortly following a TEVAR or EVAR has not been addressed before. One study suggested that this problem could at least in some cases be attributed to the dislodgement of an aortic atheroma, either spontaneously or more likely caused by intervention device, resulting in an aorto-cerebral embolic stroke [30]. This is supported by the cortical brain infarctions of LVO shown on most of the CT brains and CTAs of PIS patients in this series. Although the presence of an aortic arch atheroma and its grade were not independent risks of PIS in either intervention in this study, they were found to be significant risks in TEVAR-related PIS in the univariable analysis. This may imply that a plaque ruptured from the aortic arch atheroma could cause TEVAR-related PIS. Furthermore, cerebral hypoperfusion, found in four PIS cases (three TEVAR- and one EVAR-related), as shown by watershed infarctions on the CT brains could be another possible mechanism of PIS due to a large volume of intra-intervention blood loss, which was an independent risk of TEVAR-related PIS in this study.

Three vertebrobasilar or posterior circulation (PC) strokes were found in the EVAR-related PIS patients compared with only one case of TEVAR-related PIS in this study. The higher rate of PIS in EVAR than in TEVAR noted in this study draws attention and needs explanation since the locations of aortic pathologies in the patients who underwent EVAR were far from the aortic arch, where the supra-aortic arteries supplying blood to the brain originate. We speculate that the tip of an intervention device wire may have accidentally passed beyond the abdominal aortic pathology to reach the aortic arch or even the supra-aortic artery ostia causing an arterio-cerebral embolism because of the invisibility of the device wire tip on radiologic fluoroscopy while performing an EVAR. A PC stroke has been known more detrimental than a carotid (anterior circulation, AC) stroke in aortic EVI [7,32]. We also found that intervention time was an independent risk factor for PIS in EVAR. The finding that long intervention time increased the risk of PIS in one aortic intervention study supports our finding. [32].

The coverage of LSCA with concomitant carotid-subclavian or other supra-aortic revascularization has been encouraged by many interventional studies, including the practice guidelines issued by The Society of Vascular Surgery, to prevent a PC stroke during TEVAR [7,9,13,31,33,34], despite a lack of strong evidence to support the practice. LSCA coverage with concomitant supra-aortic revascularization was performed in all cases of TEVAR in our institution during the study period when it was indicated; however, it was not performed in any cases of EVAR-related PIS. Hence, we conclude that a long intervention time, non-visualized device tip with accidentally ruptured aortic arch atheroma, and lack of LSCA protection procedures contributed to the higher rate of EVAR-related PIS found in the current study.

Post-intervention hyperglycemia was another independent risk factor for PIS in the EVAR cases, while it was a dependent risk factor in the TEVAR cases. Because three (two TEVAR and one EVAR) of eleven (seven TEVAR and four EVAR) PIS cases with post-intervention hyperglycemia were known to have DM before the intervention, we speculate that the post-intervention hyperglycemia in our PIS cases was stress-induced hyperglycemia due to the complexity of the cases, which was reflected by the significantly long intervention time in the EVAR cases. Post-intervention hyperglycemia is a plausible cause of PIS or can even worsen stroke outcome. A study on carotid stenting reported that an HbA1C level > 7.0% in diabetic patients was an independent risk factor for 30-day peri-operation stroke [35]. No reports of hyperglycemia as an independent risk factor of PIS in TEVAR or EVAR were found in the literature review. Post-intervention AF is also worth consideration as a possible risk of PIS, especially in EVAR cases. Although AF did not have a significant independent association with PIS, it was a dependent risk for EVAR-related PIS in this study.

Because the PISs in this study were detected by clinical observation during the recovery phase shortly following the interventions when the post-intervention patients could not cooperate well with a clinical assessment, it was impossible to pinpoint the actual onset times of the PISs and possible the delay in the activation of acute stroke management process. Hence, most of the emergency CT brains at the time of hospital stroke team notification showed subacute and large area of brain infarctions representing significant and irreversible ischemic damage of the brain parenchyma. For this reason, PIS patients in this series were not eligible for thrombolytic therapy or cerebral thrombectomy. Consequently, significantly longer hospitalization and more unfavorable outcomes resulted. It is accepted that if appropriate acute ischemic stroke treatments can be applied timely, favorable neurological outcomes are achievable [36].

This was a single center experience of PIS associated with TEVAR and EVAR. The limited sample size results in less power for generalization. Nonetheless, since the study had a high PIS-to-non-PIS case ratio for strengthening the statistical analysis and the non-PIS cases were randomly selected from the patient pool, we believe that the findings could be applied in some medical settings similar to ours. We realize that the inclusion of a large numbers of the variables from pre- to post-intervention in this study may result in an overfitting effect in statistical analysis. Nevertheless, these variables were considered as possibly significant risks of PIS related to the both interventions from the discussion among the authors because most of the PISs occurred very early after the interventions in this study.

Because of the non-significant differences between the case and control groups in regard to pre-intervention cerebral thrombo-embolic risks, diagnosis of aortic pathologies including their complications (ruptured vs. unruptured), states of performing the interventions (emergency or elective), methods of anesthesia provided, and the operators and their technique used in this study, it is likely that the risks of PIS identified were independent of the aortic pathology, patient characteristics, and operator skill. However, we accept that it is not unusual that the sharing of operator expertise and techniques occurs in this level of study institution. Future multi-center studies enrolling a larger number of cases and including different levels of service settings will be useful to report the more generalizable study results.

## 5. Conclusions

PIS as a complication of TEVAR or EVAR is associated with high morbidity and mortality. Operator skill and techniques have been rapidly developing in the recent years, and studies on preventive strategies for PIS associated with the interventions have also been encouraging and progressive. Careful planning of the intervention procedures and applying the proved strategies to minimize intra-intervention blood loss, shorten intervention time, close monitor and properly treat peri-intervention hyperglycemia are required.

## Figures and Tables

**Table 1:** Pre-, intra-, and post-intervention risks of TEVAR-related peri-intervention strokes by univariable analysis and multivariable analysis.

	Univariable Analysis			Multivariable Analysis	
Variable	PIS (*n* = 9)	Non-PIS (*n* = 36)	*p* Value	OR (95% CI)	*p* Value
*Pre-intervention*					
Sex, male	7 (77.8)	23 (63.9)	0.695 _1_		
Age, years, mean (SD)	71.8 (8.8)	62.5 (13.2)	0.053 _2_ *	2.38 (1.78–2.98)	0.067
BMI, kg/m^2^, mean (SD)	20.8 (2.3)	23.3 (3.9)	0.704 _2_		
Hypertension	6 (66.7)	26 (72.2)	0.793 _1_		
Diabetes mellitus	1 (11.1)	3 (8.3)	0.267 _3_		
Dyslipidemia	6 (66.7)	15 (41.7)	0.698 _1_		
Renal insufficiency	2 (22.2)	12 (33.3)	0.409 _3_		
Peripheral arterial disease	0 (0.0)	2 (5.5)	0.614 _3_		
Coronary artery disease	2 (22.2)	5 (13.9)	0.469 _3_		
Atrial fibrillation	0 (0.0)	2 (5.5)	0.651 _3_		
Previous stroke/TIA	2 (22.2)	6 (16.6)	0.284 _3_		
COPD	1 (11.1)	3 (8.3)	0.793 _3_		
Antiplatelet(s) used	4 (44.4)	12 (33.3)	0.569 _1_		
Anticoagulant(s) used	0 (0.0)	1 (2.7)	0.613 _3_		
Smoking	6 (66.7)	16 (44.4)	0.014 _1_ *	1.34 (0.14–12.98)	0.916
Previous cardiac surgery	0 (0.0)	8 (22.2)	0.179 _3_		
Pre-intervention inotropic drug	0 (0.0)	3 (8.3)	0.370 _3_		
Pre-int SBP mmHg, mean (SD)	120 (15.64)	130 (18.12)	0.148 _2_		
Site of maximal atheromatous thickness			0.770 _1_		
Aortic arch	3 (33.3)	13 (36.1)			
Descending thoracic aorta	0 (0.0)	3 (8.3)			
Descending abdominal aorta	3(33.3)	8 (22.2)			
Grading of atheroma thickness			0.219 _1_		
I	0 (0.0)	7 (19.4)			
II	1 (11.1)	9 (25.0)			
III	3 (33.3)	9 (25.0)			
IV	5 (55.6)	10 (27.8)			
Aortic arch atheroma	7 (77.8)	35 (97.2)	0.036 _1_ *	0.10 (0.01–1.26)	0.288
Grading of aortic arch atheroma			0.032 _1_ *	0.18 (0.04-0.73)	0.169
I	0 (0.0)	8 (22.2)			
II	0 (0.0)	9 (25.0)			
III	3 (33.3)	13 (36.1)			
IV	4 (44.4)	5 (13.9)			
Diagnosis			0.659 _1_		
Aortic aneurysm	8 (88.8)	27 (75)			
Aortic dissection	1 (11.1)	9 (25)			
Location of aorta			0.827 _1_		
Aortic arch	2 (22.2)	9 (25)			
Descending aorta	8 (88.8)	31 (86.1)			
Ruptured aortic aneurysm	2 (22.2)	4 (11.1)	0.537 _3_		
*Intra-intervention*					
Surgeon			0.885 _1_		
No. 1	3 (33.3)	8 (22.2)			
No. 2	2 (22.2)	8 (22.2)			
No. 3	2 (22.2)	8 (22.2)			
No. 4	2 (22.2)	12 (33.3)			
Emergency surgery	6 (66.7)	24 (66.7)	> 0.999 _1_		
General anesthesia			> 0.999 _1_		
Target control infusion	9 (100.0)	33 (91.7)			
Total intravenous anesthesia	0 (0.0)	3 (8.3)			
Intervention time, min, median (IQR)	420 (142–570)	175 (121–288)	0.047 _4_ *	2.07 (1.87–2.27)	0.402
Hypotension,	8 (88.9)	26 (72.2)	0.416 _1_		
Blood loss, mL, median (IQR)	1400 (600–1600)	360 (250–1900)	0.016 _4_ *	1.99 (1.88–21.12)	<0.001 *
LSCA coverage and bypass	5 (55.5)	12 (33.3)	0.265 _1_		
Chimney graft used	0 (0)	3 (8.3)	>0.999 _3_		
Hypoglycemia	0 (0.0)	0 (0.0)	>0.999 _3_		
Hyperglycemia	2 (22.2)	1 (2.8)	0.097 _3_ *	10 (0.79, 126.03)	0.289
Atrial fibrillation	1 (11.1)	1 (2.8)	0.364 _3_		
*Post-intervention*					
Hypotension	2 (22.2)	3 (8.3)	0.258 _3_		
Atrial fibrillation	1 (11.1)	0 (0.0)	0.200 _3_		
Hypoglycemia	0 (0.0)	1 (2.8)	>0.999 _3_		
Hyperglycemia	4 (44.4)	3 (8.3)	0.027 _3_ *	8.69 (1.51–50.08)	0.080
Respiratory failure	6 (66.7)	12 (33.3)	0.126 _1_		
Re-operation(s)	1 (11.1)	1 (2.8)	0.364 _3_		
Acute coronary syndrome	0 (0.0)	0 (0.0)	>0.999 _3_		
Congestive heart failure	0 (0.0)	1 (2.8)	>0.999 _3_		

Data are presented as *n* (%) unless indicated otherwise. PIS: peri-intervention stroke; BMI: body mass index; IQR: interquartile range; TIA: transient ischemic attack; COPD: chronic obstructive pulmonary disease; Pre-int: pre-intervention; SBP: systolic blood pressure; TEVAR: thoracic endovascular aortic repair; EVAR: endovascular abdominal aortic repair; min: minutes; OR: odds ratio. 1: Chi-square test, 2: Unpaired *t* test, 3: Fisher’s exact test, 4: Wilcoxon Rank Sum test. * Statistical significance with *p* < 0.1 for univariable analysis and *p* < 0.05 for multivariable analysis.

**Table 2:** Pre-, intra-, and post intervention risks of EVAR-related peri-intervention strokes by univariable analysis and multivariable analysis.

	Univariable Analysis			Multivariable Analysis	
Variables	PIS (*n* = 8)	Non-PIS (*n* = 32)	*p* Value	OR (95 CI)	*p* Value
*Pre-intervention*					
Sex, male	7 (87.5)	28 (87.5)	>0.999 _1_		
Age, years, mean (SD)	70.3 (5.2)	71.9 (8.3)	0.589 _2_		
BMI, kg/m^2^, mean (SD)	20.9 (3.4)	22.4 (3.4)	0.278 _2_		
Hypertension	7 (87.5)	27 (84.4)	0.825 _1_		
Diabetes mellitus	2 (25.0)	5 (15.6)	0.533 _3_		
Dyslipidemia	4 (50.0)	20 (62.5)	0.519 _1_		
Renal insufficiency	3 (37.5)	17 (53.1)	0.429 _1_		
Peripheral arterial disease	1 (12.5)	0 (0.0)	0.200 _3_		
Coronary artery disease	1 (12.5)	1 (3.1)	0.277 _3_		
Atrial fibrillation	0 (0.0)	1 (3.1)	0.613 _3_		
Previous stroke/TIA	3 (37.5)	0 (0.0)	0.006 _3_ *	1.03 (0.84–1.21)	0.999
COPD	2 (25.0)	3 (9.4)	0.006 _3_ *		
Antiplatelet used	6 (75.0)	15 (46.9)	0.232 _1_		
Anticoagulant used	0 (0.0)	0 (0.0)	0.241 _3_		
Smoking	5 (62.5)	17 (53.1)	>0.999 _1_	0.90 (0.22–3.67)	0.550
Previous cardiac surgery	2 (25.0)	1 (3.1)	0.090 _3_ *	10.33 (0.80–132.96)	0.190
Preintervention inotropic drug	1 (12.5)	2 (6.3)	0.096 _3_ *		
Pre-int SBP mmHg, mean (SD)	121.0 (29.3)	130.5 (17.7)	0.498 _2_		
Sites of the maximal atheromatous thickness			0.133 _1_		
Aortic arch	3 (37.5)	12 (37.5)			
Descending thoracic	1 (12.5)	0 (0.0)			
Descending abdominal aorta	1 (12.5)	12 (37.5)			
Grading of atheroma thickness			0.919 _1_		
I	0 (0.0)	1 (3.1)			
II	1 (12.5)	3 (9.4)			
III	2 (25.0)	6 (18.8)			
IV	4 (50.0)	14 (43.8)			
Aortic arch atheroma	7 (87.5)	24 (75.0)	0.449 _1_		
Grading of aortic arch atheroma			0.789 _1_		
I	0 (0.0)	1 (3.1)			
II	1 (12.5)	4 (12.5)			
III	3 (37.5)	6 (18.5)			
IV	4 (37.5)	13 (40.6)			
Diagnosis			>0.999 _1_		
Aortic Aneurysm	8 (100)	32 (100)			
Aortic dissection	0 (0.0)	0 (0.0)			
Location of abdominal aorta			0.574 _1_		
Suprarenal	0 (0.0)	1 (3.1)			
Infrarenal	8 (100.0)	28 (87.5)			
Rupture aortic aneurysm	1 (12.5)	8 (25)	0.442 _1_		
*Intra-intervention*					
Surgeon			0.310 _1_		
No. 1	4 (50.0)	9 (28.1)			
No. 2	3 (37.5)	8 (25.0)			
No. 3	0 (0.0)	8 (25.0)			
No. 4	1 (12.5)	7 (21.9)			
Emergency surgery	7 (87.5)	24 (75.0)	0.655 _1_		
General anesthesia			0.495 _1_		
Target control infusion	6 (75.0)	24 (75.0)			
Total intravenous anesthesia	2 (25.0)	8 (25.0)			
Intervention time, min, median (IQR)	300 (160–540)	160 (120–210)	0.053 _4_ *	2.16 (1.95–2.37)	0.010 *
Hypotension,	6 (75.0)	17 (53.1)	0.428 _1_		
blood loss, mL, median (IQR)	425 (125,1900)	125 (50–300)	0.130 _4_		
Chimney graft used	3 (37.5)	2 (6.2)	0.047 _3_ *	1.38 (0.42–2.56)	0.596
Hypoglycemia	0 (0.0)	0 (0.0)	>0.999 _3_		
Hyperglycemia	0 (0.0)	0 (0.0)	>0.999 _3_		
Atrial fibrillation	0 (0.0)	1 (3.1)	>0.999 _3_		
*Post-intervention*					
Hypotension	0 (0.0)	1 (3.1)	>0.999 _3_		
Atrial fibrillation	4 (50.0)	0 (0.0)	0.001 _3_ *	1.29 (0.25–22.92)	0.709
Hypoglycemia	0 (0.0)	1 (3.1)	>0.999 _3_		
Hyperglycemia	3 (37.5)	1 (3.1)	0.020 _3_ *	18.60 (1.60–216.06)	0.001 *
Respiratory failure	4 (50.0)	6 (18.8)	0.089 _1_ *	4.33 (0.84–22.47)	0.608
Re-operations	0 (0.0)	0 (0.0)	>0.999 _3_		
Acute coronary syndrome	0 (0.0)	0 (0.0)	>0.999 _3_		
Congestive heart failure	0 (0.0)	0 (0.0)	>0.999 _3_		

Data are presented as *n* (%) unless indicated otherwise. PIS: peri-intervention stroke; BMI: body mass index; IQR: interquatile range; TIA: transient ischemic attack; COPD: chronic obstructive pulmonary disease; Pre-int: preintervention; SBP: systolic blood pressure; TEVAR: thoracic endovascular aortic repair; EVAR: endovascular abdominal aortic repair; min: minutes; OR: odds ratio. 1: Chi-square test, 2: Unpaired *t* test, 3: Fisher’s exact test, 4: Wilcoxon Rank Sum test. * Statistical significance with *p* < 0.1 for univariable analysis and *p* < 0.05 for multivariable analysis.

## Data Availability

All the data and study methods were reported in the manuscript. No data were deposited in any depository sources.
